# Delineating the role of single-nucleotide polymorphism of CYP19 gene on aromatase activity in South Indian women with polycystic ovary syndrome

**DOI:** 10.1186/s43141-023-00540-7

**Published:** 2023-08-21

**Authors:** Pravesh Hegde, Shilpa S. Shetty, Prasanna Kumar Shetty, Lakshmi Manjeera, D. Prashanth Shetty, Suchetha Kumari

**Affiliations:** 1 Department of Biochemistry, KS Hegde Medical Academy, NITTE (Deemed to Be University), Deralakatte, Mangalore, 575018 Karnataka India; 2 KS Hegde Medical Academy, NITTE (Deemed to Be University), Karnataka 575018 Deralakatte, Mangalore, India; 3 Department of Obstetrics and Gynecology, KS Hegde Medical Academy, NITTE (Deemed to Be University), Deralakatte, Mangalore, 575018 Karnataka India; 4 KSHEMA Centre for Genetic Services, KS Hegde Medical Academy, NITTE (Deemed to Be University), Deralakatte, Mangalore, 575018 Karnataka India

**Keywords:** CYP19, rs2414096, Single-nucleotide polymorphism, Aromatase activity, Hyperandrogenism

## Abstract

**Background:**

Polycystic ovary syndrome is a common multifactorial endocrinopathy disorder affecting 5–15% of reproductive women worldwide. The CYP19 gene encodes key enzyme aromatase involved in androgen-to-estrogen conversion which plays a crucial role in the pathophysiology of the syndrome. Very few studies have been done in the Indian population; hence, we investigated whether CYP19 gene rs2414096 SNP is associated with PCOS and hyperandrogenism susceptibility in Karnataka women.

**Methods:**

Three-hundred subjects including 150 PCOS and 150 age-matched controls were involved in the current case–control study. Sex hormones and biochemical estimation were performed by ELISA. Sanger sequencing and PCR–RFLP were used to genotype the SNP rs2414096. Genotypic-phenotypic association was studied. Statistical analysis was performed.

**Results:**

The GG genotype was more common in patients, while the GA genotype was more common in control women. LH/FSH was significantly increased in GG genotype in PCOS when compared with AA and GA genotypes. Variations of CYP19 rs2414096 were not statistically significant with PCOS.

**Conclusion:**

CYP19 rs2414096 polymorphism was not associated with PCOS; however, the homozygous wild GG genotype may exhibit reduced aromatase activity with subsequent hyperandrogenism implicating endocrine abnormalities.

## Background

Polycystic ovary syndrome (PCOS) is a complex metabolic and heterogeneous condition seen in an estimated one in five (20%) reproductive-aged women in India [1]. It is distinguished by hyperandrogenism and chronic anovulation leading to obesity and developing polycystic ovaries which progress to infertility. Excess production of ovarian androgens is one of the major pathophysiological features of PCOS since they play a major role in normal development and reproduction [2, 3]. Clinically, hirsutism, alopecia, and/or elevated testosterone levels are signs of hyperandrogenism in females [4]. Aberrations in steroid metabolic pathways might result in an imbalance in their levels which may cause several hormone-related disorders [5]. Several enzymes are crucial for the mediation of these pathways; of which aromatase, a monooxygenase enzyme catalyzes many reactions involved in the synthesis of cholesterol, steroids, and other lipids [6].

Aromatase, a member of the cytochrome P450 enzyme family (subfamily 19), is an important steroidogenic enzyme localized in the endoplasmic reticulum and is responsible for the conversion of androgens from theca cells of the ovary to C18 estrone and E2, respectively, which is the final and rate-limiting step in steroidogenesis pathway and also been associated with circulating estrogen levels. This enzyme complex is composed of cytochrome P450 aromatase and nicotinamide adenine dinucleotide phosphate (NADPH) cytochrome P450 reductase [7, 8]. This enzyme is encoded by the CYP19A1 gene (p450arom) in the long arm of chromosome 15 (15q21.2) and spans a total of 123 kb (30-kb long coding region and 93-kb regulatory region) and hence considered as a potential candidate gene involved in PCOS pathophysiology [9]. Studies have shown that variations in women’s serum androgen concentrations within racial/ethnic groups are known to be correlated with the SNPs of the CYP19 gene. However, few studies have shown no such associations [10–12]. CYP19 rs2414096 is an intronic variant (G > A) located on intron 2 near exon 3, is responsible for hyperandrogenism, and has also been reported to be associated with PCOS [12]. Hyperandrogenosis occurs as a result of aromatase activity induced by FSH-stimulated granulosa cells, resulting in decreased serum FSH concentrations and low estradiol levels, and the condition in PCOS appears to be linked with insulin action. This reduction in aromatase activity could explain why androgen excess contributes to abnormal follicular development. Given that there is conflicting evidence linking the findings to the significance of the CYP19 gene (rs2414096) in androgen metabolic pathways, the objective of this current study was to investigate the effect of CYP19 gene polymorphism on the susceptibility of developing PCOS and hyperandrogenism in South Indian women.

## Methods

The current case–control study was approved by the institutional ethics committee. We recruited 150 women with PCOS and 150 healthy controls within 19–40 years from the department of obstetrics and gynecology and KSHEMA IVF of Justice KS Hegde Charitable Hospital, Mangalore, India. The sample size for the study was calculated by estimating the difference between two means $\frac{{N=\left({Z}_{1-\mathrm{\alpha }/2}+{Z}_{\upbeta }\right)}^{2}\left[{\mathrm{P}}_{1}\left(1-{\mathrm{P}}_{1}\right)+{\mathrm{P}}_{2}\left(1-{\mathrm{P}}_{2}\right)\right]}{{\mathrm{P}}_{1}-{\mathrm{P}}_{2}}$.

A pre-designed proforma was used to record the subject’s anthropometric, clinical, menstrual, and family history. All study participants provided written informed consent.

### Selection of subjects

PCOS women were recruited based on the 2003 Rotterdam criteria which require the presence of at least two of the following three features for diagnosis: (1) clinical manifestations or/and signs of biochemical hyperandrogenism, (2) oligovulation and/or anovulation, and (3) occurrence of polycystic ovaries on ultrasound. Women who were pregnant and had hyperandrogenic conditions like Cushing’s syndrome, late-onset congenital hyperplasia, and hyperprolactinemia were excluded from the study. Control women who had no history of infertility, clinical hyperandrogenism, and regular menstrual cycles were selected for the study.

### Anthropometric and laboratory analysis

Clinical details such as body weight, height, and waist-to-hip circumference were assessed. BMI was calculated by dividing body weight in kilograms by height in meters squared. Body weight categories (lean, normal, overweight, and obese) were defined as per the criteria of the World Health Organization (WHO).

Peripheral blood of about 5 mm was drawn by venipuncture from willing participants during the third to the fifth day of a spontaneous menstrual cycle. The serum samples were separated and stored in a deep freezer at − 20 °C, and assays were performed.

Plasma glucose was determined by the oxidase–peroxidase method with the use of a glucose analyzer. Testosterone (T), estradiol (E2), follicle-stimulating hormone (FSH), luteinizing hormone (LH), and insulin levels were performed by commercially available ELISA kits, and absorbance was read by (*Spark Tecan*) ELISA reader. The homeostatic model assessment of insulin resistance (HOMA-IR) formula was used to calculate HOMA IR:HOMA IR = fasting insulin (IU/mL) × [fasting glucose (mg/dL)]/405.

### Genotyping

**Fig. 1:**
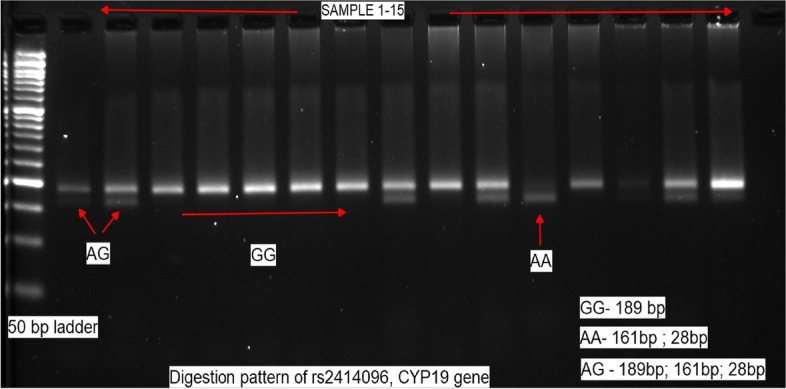
Detection of G/A polymorphism of rs2414096 CYP19 gene by restriction fragment length polymorphism after digestion with CviAII enzyme overnight at 25 °C

**Fig. 2:**
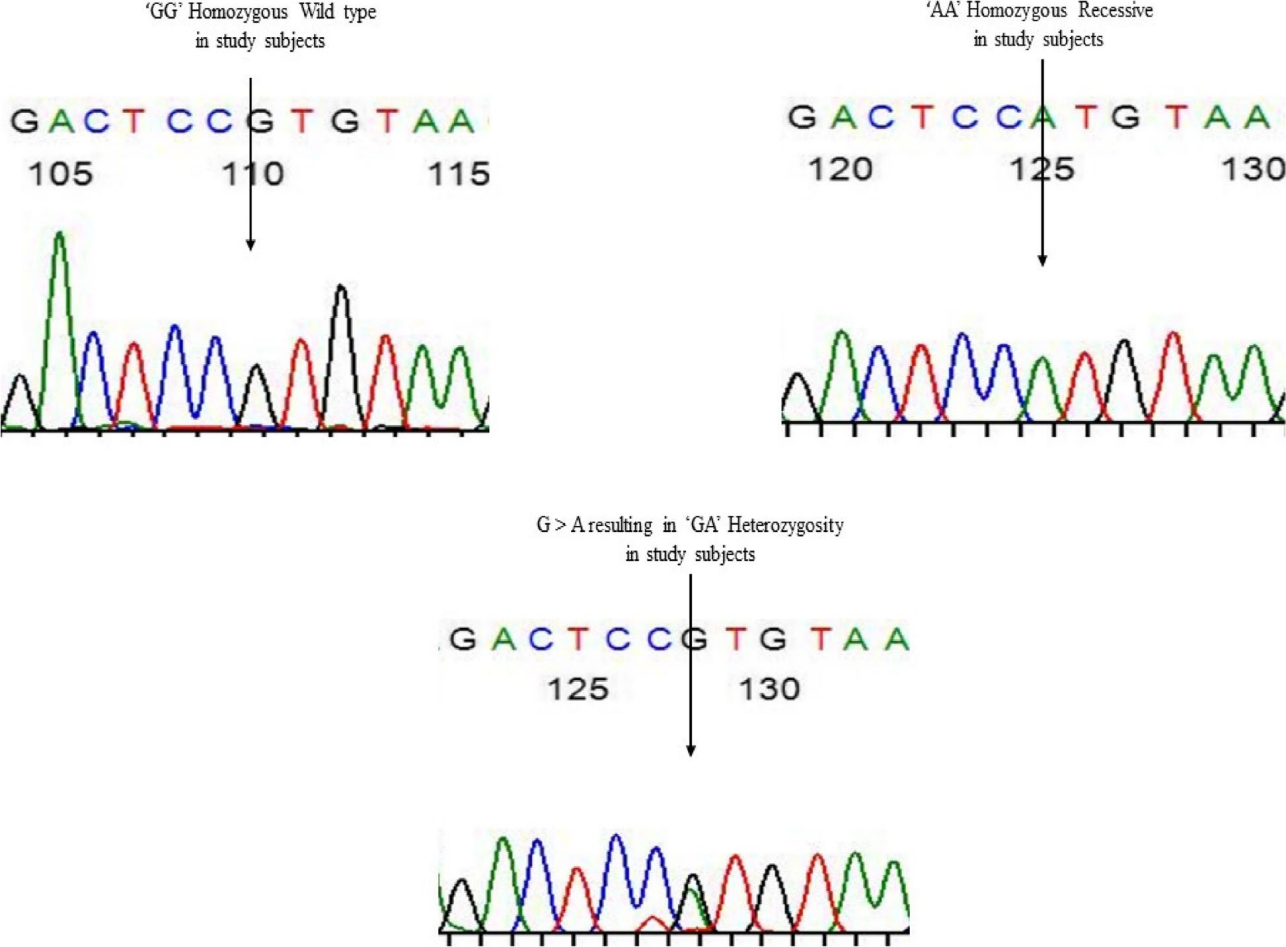
Chromatogram results by Sanger sequencing of CYP19 gene (rs2414096) substitution of G > A resulting in “GA” heterozygous in the study subjects, the arrow indicates the site of polymorphism

A commercially available kit was used to isolate DNA from blood collected in EDTA (Qiagen). A nanodrop spectrophotometer (Eppendorf) at 260 nm was used to determine the amount of DNA isolated. For determining the purity of extracted DNA, the OD 260/280 ratio was between 1.8 and 2. Genotyping of SNP rs2414096 (G > A) of the CYP19 gene for all subjects was performed by polymerase chain reaction-restriction fragment length polymorphism (PCR–RFLP) method. The sequences of the primers used were 5′-TCT GGA AAC TTT TGG TTT GAG TG-3′ (forward primer) and 5′-GAT TTA GCT TAA GAG CCT TTT CTT ACA-3′ (reverse primer). Amplification of DNA by PCR was performed in a overall volume of 25 µL with 100 ng of genomic DNA as a template in the reaction mixture and 10 µM of each primer, 10.5 µL of nuclease-free water, and 12.5 µL of 2 × RED Master Mix (Ampliqon™, Denmark) composed of Taq DNA polymerase, MgCl_2_, buffer, and dNTPs. The PCR was carried out in a MJ Mini thermal cycler (BioRad., Japan) with cycling parameters of initial denaturation at 95 °C for 5 min, amplification step consisting of 35 cycles at 95 °C for 30 s, 30 s of annealing at 53 °C, treatment at 72 °C for 30 s, and a final extension step at 72 °C for 5 min. The PCR product (189 bp) was then digested with CviAII (New England Biolabs) restriction enzyme at 25 °C overnight. Digested DNA fragments were electrophoresed in 3% agarose gel comprised of ethidium bromide and visualized under a UV transilluminator. DNA with wild-type homozygous GG genotype generated a single band of 189 bp, whereas the presence of two bands 161 bp and 28 bp indicated homozygosity for the AA genotype. The heterozygous GA genotype yielded three bands of 189 bp, 161 bp, and 28 bp (Fig. 1). Finally, selected DNA samples of genotypic data attained from RFLP analysis were randomly chosen to sequence directly for validation of rs2414096. The PCR products were purified with a DNA gel extraction kit (Norgen Biotek, Canada) and then sent to Barcode Biosciences, Bangalore, for sequencing by Sanger sequencing where similar result patterns were obtained (Fig. 2).

### Statistical analysis

Anthropometric, biochemical, and hormonal parameters between case and control were expressed as mean ± SD, where an unpaired Student *t*-test was applied for normally distributed data. Skewed data were represented as a median and interquartile range by applying Mann–Whitney *U*-test. Categorical variables were denoted as percentages, and alterations in genotypic and allelic frequencies were assessed by using chi-square analysis. To assess the relative risk of dominant, recessive, and additive models, odds ratio and 95% confidence interval were applied. ANOVA combined with Bonferroni multiple comparison for mean and Kruskal–Wallis for median was applied in the general genotype model. Differences between the groups were considered statistically significant at the level of *P* < 0.05. All analysis was performed using SPSS statistical package (IBM, version 22.0, SPSS Inc., Chicago, IL, USA).

## Results

The baseline anthropometric and biochemical characteristics of 300 study participants are presented in Table 1 where marked differences were revealed between the groups. The total mean ± SD age of PCOS and controls was 25.59 ± 5.59 and 25.12 ± 6.91. BMI and WHR were found to be statistically different between the groups (*P* = 0.000). Women with PCOS had statistically significantly higher fasting blood glucose, HOMA IR, and insulin levels (*P* = 0.000) when compared with normal controls. The levels of LH, testosterone, and LH/FSH ratio were significantly higher in PCOS compared to healthy women (*P* < 0.05). In addition, PCOS women had lower estradiol levels which were statistically significant (*P* < 0.05).

**Table 1:** Anthropometric and biochemical data among the study participants

	PCOS (N-150)	Controls (N-150)	*p*-value
BMI^a^	24.50 ± 4.87	21.66 ± 3.94	0.000**
WHR^a^	0.84 ± 0.05	0.80 ± 0.06	0.000**
FBS (mg/dL)^a^	93.09 ± 18.73	79.61 ± 15.22	0.000**
Plasma insulin (µIU/mL)^b^	17.07 (7.40–29.09)	6.26 (2.64–15.12)	0.000**
HOMA-IR^b^	3.64 (1.54–7.71)	1.17 (0.46–3.45)	0.000**
FSH (IU/L)^b^	4.94 (3.18–6.41)	4.99 (3.5–7.16)	0.244
LH (IU/L)^b^	9.49 (5.52–16.79)	7.37 (4.88–13.76)	0.046*
LH/FSH^b^	2.26 (1.44–3.7)	1.72 (1.11–2.32)	0.000**
Testosterone (nmol/L)^b^	2.75 (1.60–4.67)	1.80 (1.14–2.88)	0.000**
Estradiol (nmol/L)^b^	0.51 (0.34–0.68)	0.58 (0.35–0.78)	0.028*
E2/T (nmol/L)^b^	0.18 (0.09–0.31)	0.29 (0.17–0.52)	0.000**

*PCOS* polycystic ovary syndrome, *BMI* body mass index, *WHR* waist-hip ratio, *FBS* fasting blood sugar, *HOMA-IR* homeostatic model assessment of insulin resistance, *FSH* follicle-stimulating hormone, *LH* luteinizing hormone. Data presented as ^a^mean ± SD where Student *t*-test was applied and ^b^median (25th–75th percentile) where Mann–Whitney *U*-test was applied. *P < 0.05, **P < 0.01

The overall CYP19 rs2414096 genotypic frequency distribution in all subjects was 51.33% for GG, 5.33% for AA, and 43.33% for GA genotype. The genotypic distribution (GG, AA, GA) in the PCOS group was 79, 8, and 63 respectively which did not differ from that of the normal controls (75, 8, and 67, respectively) (*P* = 0.893). Furthermore, we found that 73.6% of the G allele was present in PCOS and 72.3% in controls. Likewise, the frequency of the A allele in PCOS patients (26.3%) was also similar to that in controls (27.6%). The frequency distribution of rs2414096 of CYP19 also verified that PCOS patients presented a slightly lower and equal distribution of GA and AA genotypes except for the GG genotype which was slightly higher as compared to control women. The genotypic distribution and allele frequencies in different models (dominant and recessive) were not detected to be statistically significant amid cases and controls (*P* = 0.644 and *P* = 1.000, respectively) (Table 2).

**Table 2:** Distribution of CYP19 rs2414096 gene polymorphism in study subjects

	PCOS (150) *N* (%)	Controls (150) *N* (%)	OR (95% *CI*)	*χ*^2^ (*p*-value)
General				
GG	79	75		0.227 (0.893)
AA	8	8		
GA	63	67		
Additive				
G	221	217	1.070 (0.7–1.5)	0.135 (0.713)
A	79	83		
Dominant				
GG	79 (52.6)	75 (50)	1.113 (0.7–1.7)	0.213 (0.644)
AA + GA	71 (47.3)	75 (50)		
Recessive				
AA	8 (5.3)	8 (5.3)	1.000 (0.3–2.7)	0.000 (1.000)
GA + GG	142 (94.6)	142 (94.6)		

Data are presented as numbers (%). A chi-square test was applied, and statistical significance was set at *P*-value < 0.05. Different genetic models (additive, dominant, and recessive models)

*OR* odds ratio, *CI* confidence interval

There was no discernible difference in the genotypic distribution of CYP19 rs2414096 based on BMI between obese and nonobese PCOS and control women, and neither group of obese women had any genotypic AA alleles (Table 3). Table 4 shows the genotype–phenotype correlation between different genotypes in PCOS women and controls (GG vs AA vs GA). Although we found no association between clinical, biochemical, and hormonal parameters, LH/FSH ratio (*P* = 0.036) was significantly increased in the GG genotype when compared with AA and GA genotypes in PCOS women. Genetic variant effects of the CYP19 gene on anthropometric, biochemical, and hormones in PCOS cases based on different models are illustrated in Table 5. In the dominant model, LH (*P* = 0.036) and LH/FSH (*P* = 0.015) were significantly increased in G allele carriers when compared to the homozygous minor A allele. No associations were observed in the recessive genotypic model.

**Table 3:** Classification of CYP19 rs2414096 allelic distribution based on BMI in polycystic ovary syndrome and control women

Group	CYP19 genotype			*p*-value
	**GG**	**AA**	**GA**	
Underweight PCOS patients	8 (5.3%)	1 (0.6%)	7 (4.6%)	0.870
Underweight control women	18 (12%)	4 (2.6%)	15 (10%)	
Normal PCOS patients	38 (25.3%)	3 (2%)	33 (22%)	0.974
Normal control women	46 (30.6%)	3 (2%)	39 (26%)	
Overweight PCOS patients	21 (14%)	4 (2.6%)	16 (10.6%)	0.472
Overweight control women	8 (5.3%)	1 (0.6%)	11 (7.3%)	
Obese PCOS patients	12 (8%)	-	7 (4.6%)	0.897
Obese control women	3 (2%)	-	2 (1.3%)	

Data are presented as numbers (%). A chi-square test was applied

**Table 4:** Anthropometric, biochemical, and serum hormones based on rs2414096 genotypes in women with PCOS and controls

Genotype	PCOS (150)				Control (150)			
	**GG (79)**	**AA (8)**	**GA (63)**	***p*-value**	**GG (75)**	**AA (8)**	**GA (67)**	***p*-value**
Age (yrs)^a^	25.9 ± 5.3	27.2 ± 5.1	24.9 ± 5.8	0.375	25.1 ± 6.7	25.8 ± 7.3	25 ± 7.1	0.949
BMI^a^	24.4 ± 4.9	23.5 ± 4.5	24.6 ± 4.9	0.815	21.6 ± 3.7	20.4 ± 3.7	21.8 ± 4.2	0.631
WHR^a^	0.84 ± 0.05	0.84 ± 0.06	0.85 ± 0.04	0.871	0.80 ± 0.06	0.78 ± 0.06	0.80 ± 0.06	0.856
FBS (mg/dL)^a^	95.5 ± 16.5	86.5 ± 20.8	90.7 ± 20.7	0.180	80.31 ± 16.59	76.12 ± 8.91	79.16 ± 14.08	0.724
Plasma insulin (µIU/mL)^b^	17.50 (7.22–34.78)	17.09 (8.96–27.41)	16.60 (5.93–26.27)	0.903	5.30 (2.59–15.24)	2.61 (1.96–15.24)	8.16 (2.82–15.08)	0.434
HOMA-IR^b^	4.01 (1.56–8.46)	3.54 (1.68–5.51)	3.60 (1.22–6.05)	0.744	0.97 (0.47–3.41)	0.51 (0.31–2.86)	1.79 (0.51–3.75)	0.466
FSH (IU/L)^b^	4.91 (3.22–6.39)	4.62 (3.53–6.74)	5.02 (2.77–6.29)	0.902	4.80 (3.53–6.58)	5.67 (2.96–7.27)	5.80 (3.39–7.30)	0.706
LH (IU/L)^b^	9.89 (5.49–17.15)	5.42 (3.90–9.39)	9.48 (6.49–17.64)	0.111	6.83 (4.56–13.69)	7.80 (4.26–15.55)	7.73 (5.56–14.09)	0.496
LH/FSH^b^	2.39 (1.45–3.66)	1.19 (0.88–1.82)	2.25 (1.46–4.08)	0.036*	1.68 (1.18–2.18)	1.85 (1.00–2.45)	1.75 (1.10–2.32)	0.949
Testosterone (nmol/L)^b^	2.78 (1.64–4.70)	2.29 (1.15–3.91)	2.42 (1.60–4.68)	0.732	1.86 (1.12–2.86)	2.16 (1.58–2.48)	1.71 (1.14–2.99)	0.902
Estradiol (nmol/L)^b^	0.51 (0.31–0.61)	0.42 (0.28–0.69)	0.54 (0.40–0.70)	0.373	0.60 (0.31–0.83)	0.43 (0.26–0.62)	0.59 (0.36–0.75)	0.197
E2/T	0.15 (0.08–0.29)	0.18 (0.08–0.39)	0.20 (0.11–0.33)	0.493	0.31 (0.14–0.59)	0.21 (0.11–0.26)	0.29 (0.19–0.46)	0.381

Values expressed as ^a^mean ± SD and.^b^median (25th–75th percentile). One-way ANOVA (parametric), and Kruskal-Wallis (non-parametric) statistical test was applied. *P < 0.05

**Table 5:** Association of CYP19 SNP rs2414096 with anthropometric, biochemical, and serum hormones in women with PCOS based on genotypic models

	General model*			*p*-value	Dominant model#		*p*-value	Recessive model#		*p*-value
	**GG [79]**	**AA **[8]	**GA [63]**		**GG [79]**	**AA + GA [71]**		**AA **[8]	**GG + GA [142]**	
Age (yrs)^a^	25.9 ± 5.3	27.2 ± 5.1	24.9 ± 5.8	0.375	25.9 ± 5.3	25.18 ± 5.81	0.397	27.25 ± 5.1	25.5 ± 5.5	0.387
BMI^a^	24.4 ± 4.9	23.5 ± 4.5	24.6 ± 4.9	0.815	24.4 ± 4.9	24.53 ± 4.87	0.937	23.5 ± 4.5	24.5 ± 4.9	0.552
WHR^a^	0.84 ± 0.05	0.84 ± 0.06	0.85 ± 0.04	0.871	0.84 ± 0.05	0.85 ± 0.05	0.884	0.84 ± 0.06	0.85 ± 0.05	0.650
FBS (mg/dL)	95.5 ± 16.5	86.5 ± 20.8	90.7 ± 20.7	0.180	95.5 ± 16.5	90.25 ± 20.65	0.080	86.50 ± 20.84	93.46 ± 18.61	0.308
Plasma insulin (µIU/mL)^b^	17.50 (7.2–34.7)	17.09 (8.9–27.4)	16.60 (5.9–26.2)	0.903	17.50 (7.2–34.7)	16.74 (7.91–26.27)	0.654	17.09 (8.96–27.41)	17.07 (7.13–30.09)	0.151
HOMA-IR^b^	4.01 (1.56–8.4)	3.54 (1.68–5.5)	3.60 (1.2–6.05)	0.744	4.01 (1.56–8.4)	3.60 (1.48–6.01)	0.444	3.54 (1.68–5.51)	3.71 (1.54–7.72)	0.795
FSH (IU/L)^b^	4.91 (3.2–6.39)	4.62 (3.53–6.7)	5.02 (2.7–6.29)	0.902	4.91 (3.2–6.39)	5.02 (2.93–6.45)	0.888	4.62 (3.53–6.74)	4.94 (3.09–6.33)	0.701
LH (IU/L)^b^	9.89 (5.4–17.1)	5.42 (3.9–9.39)	9.48 (6.4–17.6)	0.111	9.89 (5.4–17.1)	8.93 (5.61–16.25)	0.578	5.42 (3.90–9.39)	9.61 (5.98–17.21)	0.036*
LH/FSH^b^	2.39 (1.45–3.6)	1.19 (0.88–1.8)	2.25 (1.4–4.08)	0.036*	2.39 (1.45–3.6)	2.09 (1.24–3.81)	0.765	1.19 (0.88–1.82)	2.36 (1.45–3.79)	0.015*
Testosterone (nmol/L)^b^	2.78 (1.64–4.7)	2.29 (1.15–3.9)	2.42 (1.6–4.68)	0.732	2.78 (1.64–4.7)	2.42 (1.60–4.59)	0.572	2.29 (1.15–3.91)	2.75 (1.60–4.68)	0.499
Estradiol (nmol/L)^b^	0.51 (0.31–0.6)	0.42 (0.28–0.7)	0.54 (0.4–0.7)	0.373	0.51 (0.31–0.6)	0.52 (0.39–0.70)	0.272	0.42 (0.28–0.69)	0.51 (0.35–0.68)	0.569
E2/T (nmol/L)^b^	0.15 (0.08–0.3)	0.18 (0.08–0.4)	0.20 (0.11–0.3)	0.493	0.15 (0.08–0.2)	0.20 (0.10–0.33)	0.257	0.15 (0.10–0.20)	0.18 (0.09–0.31)	0.947

Values expressed as ^a^mean ± SD and.^b^median (25th–75th percentile). *One-way ANOVA (parametric), Kruskal-Wallis (non-parametric) statistical test was applied for the general model. #Student *t*-test for dominant and recessive genotype model. *P < 0.05

## Discussion

Polycystic ovary syndrome (PCOS) is the most prevalent endocrine condition predominantly in South Asian females [13]. Based on works of literature, PCOS is a complex disorder affecting the reproductive and metabolic system of one in seven women of childbearing age worldwide [14]. In India, the prevalence of PCOS is considered to be a little higher than in other Asian countries [15, 16]. Despite the growing number of emerging PCOS patients, patients often do not seek care due to a lack of awareness. Nonetheless, few studies on androgens in women’s health, particularly at the genetic level, have been conducted [11]. Several genes have previously been investigated for their relationship to PCOS, including the CYP19 gene which codes for enzymes involved in ovarian steroidogenesis and is hence considered to be a potential candidate gene in the pathophysiology of the syndrome, and their gene variants are thought to play a role in the development of hyperandrogenism in PCOS. Single-nucleotide polymorphism of rs2414096 has been linked with aromatase activity in women with PCOS with variable outcomes. The aromatase enzyme is a single product of the CYP19 gene that facilitates the conversion of androgens like testosterone and androstenedione to estrone and estradiol (E2) in the gonadal and extragonadal tissues. Aromatase deficiency has been reported in several people which might lead to excess production and metabolism of androgens, namely hyperandrogenism in PCOS when compared with healthy individuals [17]. Not many reports on single-nucleotide polymorphism have been reported for PCOS susceptibility from the region of southern India; hence, the present study was examined to understand the possible role of rs2414096 of CYP19 gene and its association with hyperandrogenism in women with PCOS among the residents from the state of Karnataka, India.

In the current study, we observed that PCOS was more common among reproductive-aged women specifically between 25 and 30 years, which advances to develop secondary infertility. For obvious reasons, obesity is more common among PCOS patients with higher BMI and waist-to-hip ratio projecting in the abdominal area which sheds light on problems linked with it [18]. According to reports, the Asian population has more fat deposition, with 35–80% of PCOS women being overweight [19]. Our findings revealed a statistically significant link between BMI and PCOS (*P* = 0.000) which was per a study also conducted in South India [20]. Similar results were also detected by Kaur et al. in North Indian women (*P* = 0.000) [21]. Based on our data, PCOS is manifested by a pattern of hormonal imbalance with increased levels of serum insulin, luteinizing hormone, and testosterone accompanied by reduced estradiol and FSH levels. LH/FSH ratio was also elevated, depending upon monthly menstrual cycle, obesity, or age which is typical in PCOS and was also consistent with previous studies [9, 22]. In addition, the E2/T ratio was significantly lower in PCOS than in non-PCOS controls, according to our findings (*P* = 0.000) which are consistent with studies conducted in the Chinese and Mexican population [19, 23] suggesting reduced aromatase activity may be the main reason behind excess androgens in PCOS women.

In the current study, the GG genotype frequency was higher in PCOS than in the control group, and vice versa. The GA genotype was more prevalent in controls than in women with PCOS. These results were inconsistent with a study conducted on Pakistani Punjabi women that revealed “AA” and “GA” were more prevalent in PCOS patients as compared to controls [9]. Furthermore, when we calculated the individual allelic frequencies between the groups, we found the frequency of the “G” allele was slightly higher in PCOS, and vice versa “A” allele was higher in controls. There were not any significant differences in the individual allelic distribution in cases vs controls. As a result, the presence of the “G” allele made them more prone to developing PCOS, while carriers of the “A” allele were less common in PCOS than in controls, implying that the presence of the “A” allele could be linked with the aromatase activity responsible for normal androgen levels, which can protect the ovaries from developing PCOS. According to the results of this study, no significant association between PCOS and the CYP19 rs2414096 polymorphism was observed (*P* = 0.893). The results of the present study were not following a study also conducted in South India by Reddy et al. [24] which showed a positive association but correlated with a North Indian population-based study [21]. A positive association of rs2414096 was also observed by Petry et al. [25] in women from Barcelona and Oxford, while studies conducted in the Iraqi and Chinese population also revealed that variations in aromatase gene were associated with features of hyperandrogenism in PCOS [8, 26]. Based on BMI, the GG genotype was observed more in PCOS than in control women, particularly in the obese subgroup. Likewise, the frequency of the AA genotype was similar in both PCOS and controls, higher in overweight PCOS subjects, while no AA genotype was observed in the obese subgroup. Contrarily, both subgroups of control women had a higher frequency of the GA genotype than PCOS patients. Both obese and nonobese PCOS and control women shared the same frequency of these genotypes, with no discernible differences. Since there were not any significant differences in the genotypic distribution, this possibly indicates that the SNP of rs2414096 in the CYP19 gene might not be associated with PCOS. Since rs2414096 is an intron, it will not have a direct affect on the aromatase protein sequence, and hence, this polymorphism may not be a direct cause of PCOS in our ethnicity. However, there may be some functional variants with strong linkage disequilibrium that contribute to PCOS in our ethnicity.

Activity of the enzyme aromatase can be induced by FSH, which shows a positive correlation with the estradiol level. So, through negative feedback, a decreased E2 level can increase FSH production [3]. This correlated with our observation where the concentration of FSH in the GG genotype showed decreased aromatase activity in comparison with the other genotypes. Furthermore, when we carried out the association of the CYP19 gene based on genotypic models with various parameters, we found that the dominant model exhibited significantly increased LH (*P* = 0.036) and LH/FSH ratio (*P* = 0.015) in the GG genotype when compared to the minor allele. Increased LH/FSH can induce androgen level and reduces the expression of estrogen, while increased androgen and decreased estrogen, in turn, aggravate LH/FSH via failure of dominant follicles, causing a vicious cycle. Also, PCOS women of the GG genotype had higher plasma insulin and HOMA-IR which was consistent with a study among the Kashmiri population, and findings by Sowers and colleagues, who had concluded a positive association of rs2414096 SNP of the CYP19 gene with hyperandrogenism in PCOS women [11, 27]. The estradiol levels in PCOS and controls were not significantly different between the three genotypes. This could be due to the hypothalamic-pituitary-ovarian axis’s negative feedback regulation. In PCOS patients, testosterone levels were higher in the homozygous GG genotype than in the other two genotypes, which may be due to lower aromatase activity in the homozygous GG genotype given that the testosterone level was high enough. In controls, however, the three genotypes of testosterone were comparable. Because CYP19 mediates the conversion of androgens to estrogens, the E2/T ratio offers crucial information regarding aromatase activity, suggesting that the ratio may be a potential indicator of aromatase activity [26]. The E2/T ratio in the current study was lower in GG genotype than that of the other two genotypes suggesting reduced aromatase activity in PCOS women. This may be due to the increased levels of testosterone in the PCOS follicular fluid resulting in the reduced expression of the aromatase enzyme in the luteinized granulosa cells. To the best of our knowledge, the association observed in the current study between genotypic models and various parameters in PCOS has not been documented previously. Zhang and colleagues concluded that hyperandrogenism is caused by inhibited aromatase activity, whereas Jin et al. proposed that the AA genotype of SNP rs2414096 could affect aromatase function, resulting in increased levels of estradiol and the development of PCOS [26, 28].

## Conclusion

In conclusion, this is the first of a kind study conducted on women from the state of Karnataka, India, to assess the role of SNP rs2414096 of the CYP19 gene and its association with hyperandrogenism manifestations. We found the SNP rs2414096 of the CYP19 gene was not associated with PCOS but showed a positive association with PCOS hyperandrogenism suggesting that CYP19 may be a genetic modifier rather than a major genetic determinant of the PCOS phenotype. Biochemical and androgen levels were increased in the wild allele than the variant allele signifying CYP19 allele variants impart a defensive role in the ovary as well as on symptoms of hyperandrogenism, indicating that the wild allele may be implicated in endocrine anomalies in Karnataka women with PCOS. This might be the result of variations in different populations attributable to various racial and geographic differences. Further functional studies and identification of genetic markers are required to analyze potential factors for a complex condition like polycystic ovary syndrome.

## Data Availability

Data is made available on request.

## Abbreviations

BMI: Body mass index
CYP19: Cytochrome P450, Family 19
DNA: Deoxyribonucleic acid
E2: Estradiol
E2/T: Estradiol to testosterone ratio
EDTA: Ethylene diamine tetra-acetic acid
ELISA: Enzyme-linked immune sorbent assay
FBS: Fasting blood sugar
FSH: Follicle-stimulating hormone
HOMA-IR: Homeostatic model assessment of insulin resistance
LH: Luteinizing hormone
PCOS: Polycystic ovary syndrome
PCR: Polymerase chain reaction
RFLP: Restriction fragment length polymorphism
SNP: Single-nucleotide polymorphism
SPSS: Statistical Package for Social Sciences
WHO: World Health Organization
WHR: Waist-hip ratio
